# Supplementary material for: The genome of an apodid holothuroid (Chiridota heheva) provides insights into its adaptation to a deep-sea reducing environment
Source: Commun Biol. 2022 Mar 10;5:224. doi: 10.1038/s42003-022-03176-4 (PMC8913654; doi:10.1038/s42003-022-03176-4)
Supplement: Supplementary file 2 — Supplementary Information [file 42003_2022_3176_MOESM2_ESM.pdf]

Supporting Information for

The genome of an apodid holothurian (Chiridota heheva) provides insights into its adaptation to a deep-sea reducing environment

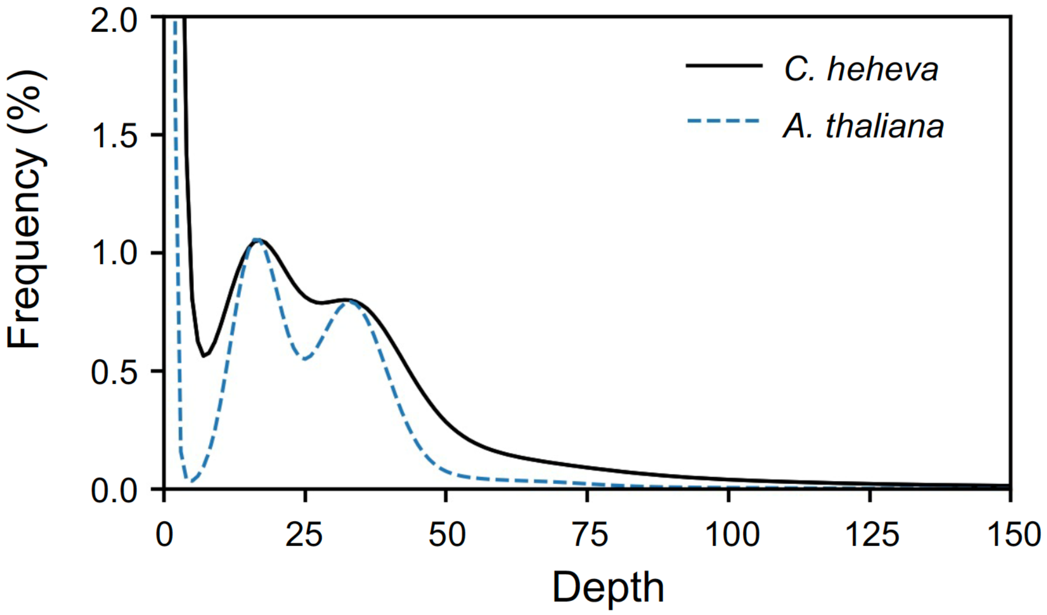

Supplementary Figure 1. Distribution of 17-mer frequency in *C. heheva* genome. The heterozygous rate and the genome size were determined based on the *k*-mer distribution. The average coverage depth is estimated to be 34 for *C. heheva* based on 150 bp paired-end Illumina reads. The heterozygosity of *C. heheva* genome was determined by fitting the distribution of *Arabidopsis thaliana*.

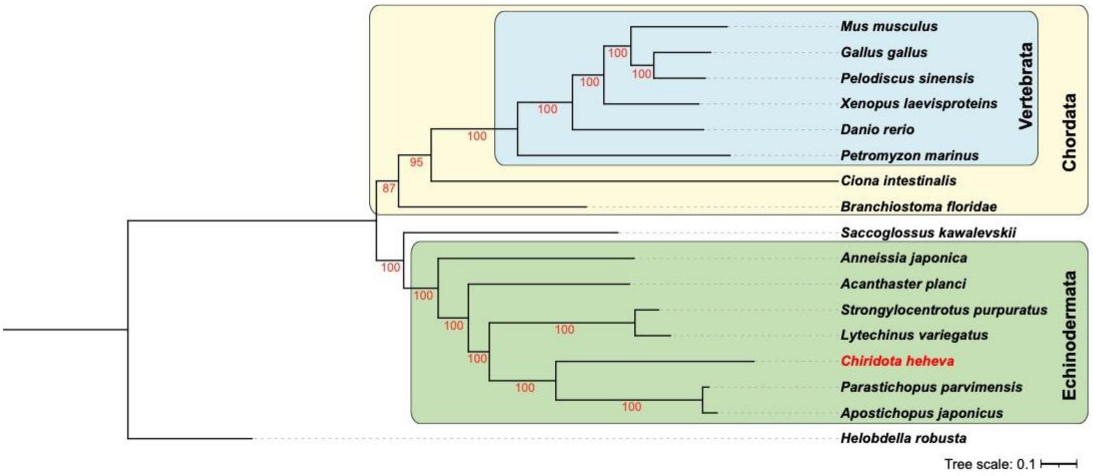

Supplementary Figure 2. The phylogenetic tree of *C. heheva* and 16 other metazoans. The tree was reconstructed with 80 single-copy orthologs using a maximum likelihood approach. The ultrafast bootstrap (UFBoots) value is listed below each of the nodes.

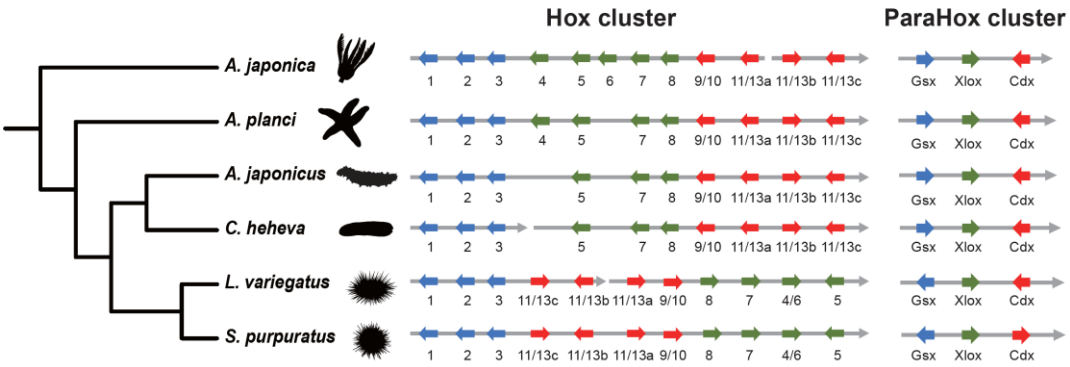

Supplementary Figure 3. Genomic organization of *Hox* and *ParaHox* gene clusters in echinoderms. *Hox* and *ParaHox* genes are indicated by arrows. The gene composition and orientation of *Hox* and *ParaHox* clusters are consistent between two holothurians (*C. heheva*, *A. japonicus*). There are some inconsistent results regarding the gene composition of *Hox* gene clusters in Echinoids. As *S. purpuratus Hox6* clusters with *A. planci Hox4* in phylogenetic analysis, it has been proposed to reclassify *S. purpuratus Hox6* as *Hox4*[1]. Therefore, we named *Hox 6* as *Hox 4/6* in the two Echinoids (*L. variegatus* and *S. purpuratus*).

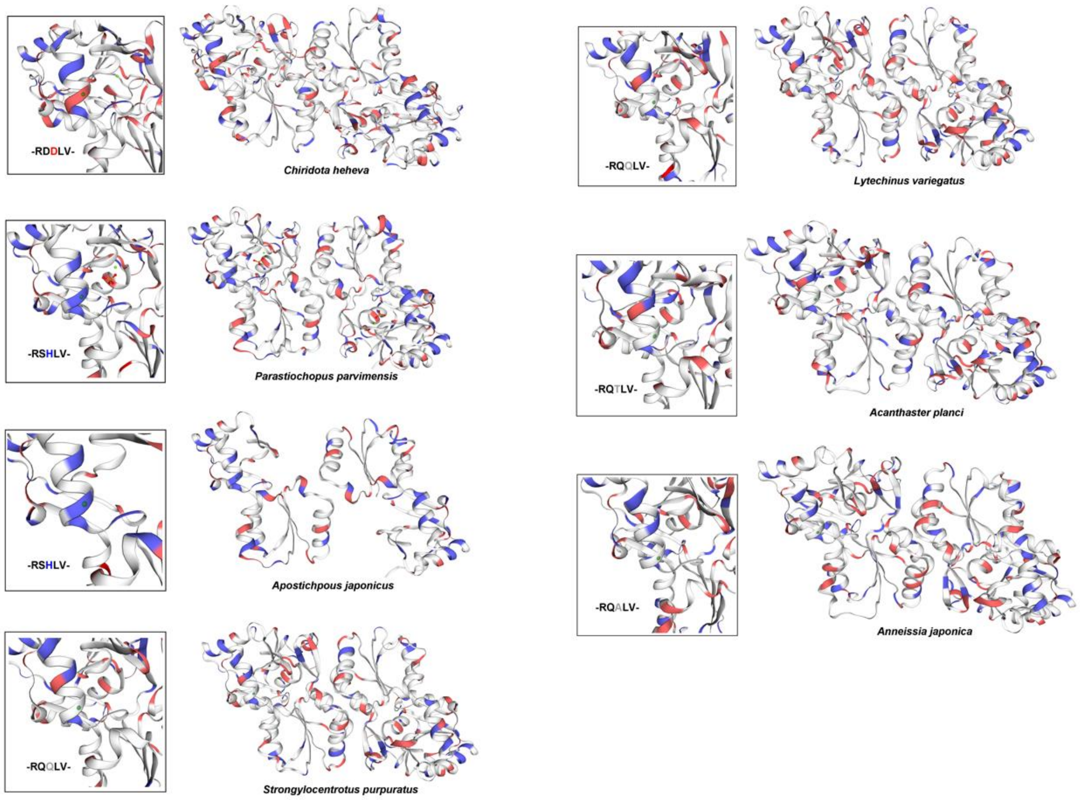

Supplementary Figure 4. Three-dimensional structure of LHPP proteins from echinoderms. Red indicates the amino acid is positively charged, blue indicates the amino acid is negatively charged, and white indicates the amino acid is uncharged. The possible amino acid substitution (position 118) of LHPP that contributed to hypoxic adaptation in *C. heheva* and cetaceans are indicated by the green dots in the embedded figures. The substitution, which is located in an α helix, does not change the conformation of LHPP.

Supplementary Table 1 Basic statistics of Nanopore reads

Total number of reads	Total number of bases (Gb)	Maximum length of reads (bp)	Minimum length of reads (bp)	Average length of reads (bp)	Depth of coverage
2,905,304	42.43	246,442	25	1460.4	34

Supplementary Table 2 Basic statistics of Illumina reads

Total number of reads	Total number of base pairs	Percentage of Q20 base pairs (%)	Percentage of Q30 base pairs (%)	GC (%)
357,304,954	49,193,650,851	96.4	90.83	38.38

Supplementary Table 3 Summary of *k*-mer analysis

***k*-mer**	***k*-mer number**	**k-mer depth**	**Genome size (bp)**	**Heterozygosity (%)**
17	41,978,881,806	34	1,234,672,994	2.0

Supplementary Table 4 BUSCO evaluation of *C. heheva* genome assembly

	C. heheva
Complete BUSCOs	855
Complete and single-copy BUSCOs	849
Complete and duplicated BUSCOs	6
Fragmented BUSCOs	27
Missing BUSCOs	72
Total BUSCO groups searched	954

Supplementary Table 5 SQUAT assessment of *C. heheva* genome assembly

	**Statistics**
No. of sequence	332,594,866
Sample size	1,000,000
Sequence length	15-140
Avgerage poorly mapped sequence	8.9%
GC content	38%

Supplementary Table 6 Summary of annotated repeats in *C. heheva* genome

	Number	Length（bp）	Percentage（%）
**Retroelements**	**277,203**	**126,998,333**	**11.47**
SINEs:	43,231	7,207,532	0.65
Penelope	9,627	3,239,963	0.29
LINEs:	210,096	107,581,691	9.72
L2/CR1/Rex	96,117	41,383,441	3.74
R1/LOA/Jockey	63,290	44,118,706	3.99
R2/R4/NeSL	125	64,396	0.01
RTE/Bov-B	28,298	11,944,523	1.08
L1/CIN4	418	66,470	0.01
LTR:	23,876	12,209,110	1.1
BEL/Pao	4,654	792,365	0.07
Gypsy/DIRS1	17,877	11,065,063	1
Retroviral	1,294	295,882	0.03
**DNA transposons：**	**79,045**	**33,585,526**	**3.03**
hobo-Activator	36,285	9,130,721	0.82
Tc1-IS630-Pogo	849	286,365	0.03
PiggyBac	158	81,861	0.01
Tourist/Harbinger	970	346,863	0.03
Other (Mirage,P-element,Transib)	3,621	539,664	0.05
**Rolling circles**	**34,565**	**9,570,594**	**0.86**
**Unclassified:**	**1,745,539**	**424,910,357**	**38.39**
**Small RNA:**	**7,394**	**1,286,798**	**0.12**
**Satellites:**	**6,112**	**1,122,725**	**0.1**
**Simple repeats:**	**52,606**	**26,896,646**	**2.43**

Supplementary Table 7 Transposable element composition in *C. heheva* and other echinoderm genomes

	Chiridota heheva (1,107 Mb)		*Apostichopus japonicus* (952 Mb)[2]		*Parastichopus parvimensis* (873 Mb)[3]		*Strongylocentrotus purpuratus* (991 Mb)[4]		*Lytechinus variegatus* (1,061 Mb)[3]		*Acanthaster planci* (384Mb)[5]		*Anneissia japonica* (553 Mb)[6]	
	Length (Mb)	Rate (%)	Length (Mb)	Rate (%)	Length (Mb)	Rate (%)	Length (Mb)	Rate (%)	Length (Mb)	Rate (%)	Length (Mb)	Rate (%)	Length (Mb)	Rate (%)
DNA	33.59	3.03	34.56	3.52	10.31	1.18	105.74	10.67	211.9	19.97	40.12	10.45	9.56	1.62
LTR	12.21	1.10	9.63	0.98	2.71	0.31	12.04	1.22	32.56	3.07	14.82	3.86	5.62	0.95
LINE	107.58	9.72	20.33	2.07	15.57	1.78	37.88	3.82	54.94	5.18	18.28	4.76	27.16	4.61
SINE	7.21	0.65	5.89	0.60	2.57	0.29	15.12	1.53	32.4	3.05	4.22	1.10	6.07	1.03
RNA	1.29	0.12	0.76	0.08	3.25	0.4	0.16	0.02	0.15	0.01	26.17	6.82	1.51	0.26
Tandem Repeat	28.02	2.53	19.34	1.97	1.34	0.16	40.23	4.06	25.31	2.39	3.41	0.89	1.95	0.33
Other	9.57	0.86	12.21	1.24	0.58	0.07	5.74	0.58	48.61	4.58	13.61	3.54	1.08	0.18
Unclassified	424.91	38.39	151.26	15.39	181.87	20.83	135.2	13.64	18.69	0.02	5.17	1.35	215.6	36.56
Total	624.38	56.40	253.98	26.68	218.2	25.02	352.11	35.54	424.56	38.27	125.8	32.77	268.55	45.54

Supplementary Table 8 Gene features of *C. heheva* and other echinoderms

	Gene counts	Average gene length (bp)	Average CDS length (bp)	Average exons per gene	Average exon size (bp)	Average intron size (bp)
C. heheva	36,527	14,943	1,496	5.37	277	2,859
A. japonicus	29,451	7,722	1,324	7.40	202	1,134
S. purpuratus	27,750	13,690	2,143	8.90	282	1,217
L. variegatus	28,094	18,033	1,062	5.30	198	1,527
A. planci	24,747	16,844	1,375	6.80	203	1,161

Supplementary Table 9 Summary of NLR genes in *C. heheva*

NLR	Domain organization
ChiHeh_gene29120	Death, NACHT, Death
ChiHeh_gene12421	DED, NACHT
ChiHeh_gene26187	NACHT
ChiHeh_gene10961	DED, DED, NACHT
ChiHeh_gene15903	NACHT
ChiHeh_gene26414	DED, NACHT
ChiHeh_gene10067	DED, DED, NACHT
ChiHeh_gene35009	DED, DED, NACHT
ChiHeh_gene30081	Death, NACHT
ChiHeh_gene12883	DED, NACHT
ChiHeh_gene25938	Death, NACHT
ChiHeh_gene1054	NACHT
ChiHeh_gene10777	Death, NACHT
ChiHeh_gene19441	NACHT
ChiHeh_gene28117	DED, DED, NACHT
ChiHeh_gene10117	NACHT
ChiHeh_gene31453	DED, DED, NACHT
ChiHeh_gene10309	DED, DED, NACHT
ChiHeh_gene34531	Death, NACHT
ChiHeh_gene14426	DED, NACHT
ChiHeh_gene8030	NACHT
ChiHeh_gene12018	NACHT, LRR_8, LRR_8, LRR_4
ChiHeh_gene4819	Death, NACHT, DDE_Tnp_1_7
ChiHeh_gene20564	NACHT
ChiHeh_gene3835	NACHT
ChiHeh_gene3488	NACHT
ChiHeh_gene7016	NACHT
ChiHeh_gene3262	DED, DED, NACHT
ChiHeh_gene7544	NACHT
ChiHeh_gene7545	DED, NACHT
ChiHeh_gene26021	DED, DED, NACHT
ChiHeh_gene10245	V-set, V-set, C2-set_2, NACHT
ChiHeh_gene12145	NACHT
ChiHeh_gene11999	NACHT
ChiHeh_gene30189	Death, NACHT
ChiHeh_gene33098	NACHT
ChiHeh_gene2291	V-set, Ig_3, NACHT
ChiHeh_gene32597	NACHT, ZU5
ChiHeh_gene17853	DED, NACHT
ChiHeh_gene25299	NACHT
ChiHeh_gene25442	NACHT
ChiHeh_gene25963	V-set, C2-set_2, NACHT
ChiHeh_gene29023	DED, NACHT, DED
ChiHeh_gene7279	NACHT
ChiHeh_gene18722	NACHT

ChiHeh_gene1587	NACHT
ChiHeh_gene7919	NACHT
ChiHeh_gene23708	NACHT
ChiHeh_gene7425	NACHT
ChiHeh_gene16374	DED, DED, NACHT
ChiHeh_gene19652	Death, NACHT
ChiHeh_gene3439	V-set, V-set, C2-set_2, NACHT, zf-B_box, zf-B_box
ChiHeh_gene22493	NACHT, NLRC4_HD2

Supplementary Table 10 Gene families that are contracted in *C. heheva* compared to other echinoderms

PANTHER ID	***Annotation***	**C. heheva**	**A. japonicus**	**P. parvimensis**	**S. purpuratus**	**L. variegatus**	**A. planci**	**A. japonica**
PTHR42743	Amino-Acid Aminotransferase	2	2	2	1	1	13	16
PTHR14002	Endoglin/Tgf-β Receptor Type III	1	2	2	1	1	5	23
PTHR14948	NG5	1	3	5	1	1	2	11
PTHR10578	S-2-Hydroxy-Acid Oxidase-Related	1	2	4	3	2	2	3
PTHR11733	Zinc Metalloprotease Family M13 Neprilysin-Related	0	1	1	1	1	11	2
PTHR22930	Uncharacterized	0	1	2	0	0	1	10
PTHR10974	Uncharacterized	0	1	2	2	1	1	6
PTHR43586	Cysteine Desulfurase	0	2	1	2	4	1	2
PTHR11841	Reelin	0	4	2	2	1	1	2
PTHR47968	Centromere Protein E	0	5	2	1	1	1	1
PTHR23033	β-1,3-Galactosyltransferase	0	1	2	4	1	1	1
PTHR12555	Ubiquitin Fusion Degradation Protein	0	3	1	1	1	3	1
PTHR14237	Molybdopterin Cofactor Sulfurase Mosc	0	1	4	2	1	1	1
PTHR14633	Little Elongation Complex Subunit 2	0	2	1	3	1	2	1

PTHR22367	Coiled-Coil Domain-Containing Protein 14	0	2	1	3	1	1	1
PTHR11730	Ammonium Transporter	0	3	1	2	1	1	1
PTHR19981	Talin	0	4	1	1	1	1	1
PTHR28434	Protein C3ORF33	0	1	2	3	1	1	1
PTHR31804	Mediator Of RNA Polymerase Ii Transcription Subunit	0	3	1	2	1	1	1
PTHR46315	Spermine Synthase	0	3	1	2	1	1	1
PTHR11360	Monocarboxylate Transporter	0	1	2	3	1	1	1
PTHR44442	3-Keto-Steroid Reductase	0	3	1	1	1	1	1
PTHR19964	Multiple PDZ Domain Protein	0	3	1	1	1	1	1
PTHR15741	Basic Helix-Loop-Helix Zip Transcription Factor	0	3	1	1	1	1	1
PTHR42690	Threonine Synthase Family Member	0	3	1	1	1	1	1

Supplementary Table 11 Positively selected genes (PSGs) in *C. heheva*

No.	Gene ID	Gene Name	Abbreviation	Omega
**1**	ChiHeh_gene24148	Exosome Component 8	*EXOSC8*	6.83705
**2**	ChiHeh_gene20072	Isoleucyl-TRNA Synthetase 1	*IARS1*	3.37011
**3**	ChiHeh_gene25641	Phospholysine Phosphohistidine Inorganic Pyrophosphate Phosphatase	*LHPP*	6.27734
**4**	ChiHeh_gene31948	Dynactin Subunit 1	*DCTN1*	7.06391
**5**	ChiHeh_gene2856	MutS Homolog 4	*MSH4*	2.92004
**6**	ChiHeh_gene15929	SRY-Box Transcription Factor 6	*SOX6*	9.79661
**7**	ChiHeh_gene11918	Sphingolipid Transporter 1	*SPNS1*	4.04511
**8**	ChiHeh_gene18484	Microsomal Triglyceride Transfer Protein	*MTTP*	3.39416
**9**	ChiHeh_gene35223	Poly(A)-specific ribonuclease subunit PAN2	*PAN2*	5.84083
**10**	ChiHeh_gene2338	Zw10 Kinetochore Protein	*ZW10*	2.25404
**11**	ChiHeh_gene23426	MAU2 Sister Chromatid Cohesion Factor	*MAU2*	6.00742
**12**	ChiHeh_gene22022	Small RNA 2'-O-methyltransferase	*HEN1*	22.11709
**13**	CheHeh_gene5792	Ankyrin Repeat Domain 6	*ANKRD6*	36.77157
**14**	ChiHeh_gene2437	Uncharaterized protein	-	5.84394
**15**	ChiHeh_gene7033	ER Degradation Enhancing Alpha-Mannosidase Like Protein 1	*EDEM1*	49.6507
**16**	ChiHeh_gene25677	Protein Phosphatase 4 Regulatory Subunit 2	*PPP4R2*	29.95628
**17**	ChiHeh_gene6288	TSR3 Ribosome Maturation Factor	*TSR3*	6.98563
**18**	ChiHeh_gene21377	Ribosomal RNA Processing 9	*RRP9*	7.58009
**19**	ChiHeh_gene22931	Diacylglycerol Kinase Gamma	*DGKG*	1.5207
**20**	ChiHeh_gene27209	Thioredoxin Reductase 1	*TXNRD1*	16.1724
**21**	ChiHeh_gene15719	Pyruvate Kinase M2	*PKM2*	3.24103
**22**	ChiHeh_gene816	Peptidase, Mitochondrial Processing Subunit Alpha	*PMPCA*	4.7463
**23**	ChiHeh_gene11519	Lysine Acetyltransferase 8	*KAT8*	6.79493

**24**	ChiHeh_gene5454	TBC1 Domain Family Member 20-like	*TBC1D20*	62.67164
**25**	ChiHeh_gene35171	Calpain 7	*CAPN7*	4.89478
**26**	ChiHeh_gene9027	NOP58 Ribonucleoprotein	*NOP58*	4.67224
**27**	ChiHeh_gene3731	tRNA-YW Synthesizing Protein 5-like	*TYW5*	6.51395

Supplementary References

1 Baughman, K. W. *et al.* Genomic organization of Hox and ParaHox clusters in the echinoderm, Acanthaster planci. *Genesis* **52**, 952-958, doi:10.1002/dvg.22840 (2014).

2 Li, Y. L. *et al.* Sea cucumber genome provides insights into saponin biosynthesis and aestivation regulation. *Cell Discov* **4**, doi:ARTN 2910.1038/s41421-018-0030-5 (2018).

3 Arshinoff, B. I. *et al.* Echinobase: leveraging an extant model organism database to build a knowledgebase supporting research on the genomics and biology of echinoderms. *Nucleic Acids Res*, doi:10.1093/nar/gkab1005 (2021).

4 Sea Urchin Genome Sequencing, C. *et al.* The genome of the sea urchin Strongylocentrotus purpuratus. *Science* **314**, 941-952, doi:10.1126/science.1133609 (2006).

5 Hall, M. R. *et al.* The crown-of-thorns starfish genome as a guide for biocontrol of this coral reef pest. *Nature* **544**, 231-234, doi:10.1038/nature22033 (2017).

6 Li, Y. *et al.* Genomic insights of body plan transitions from bilateral to pentameral symmetry in Echinoderms. *Commun Biol* **3**, 371, doi:10.1038/s42003-020-1091-1 (2020).
