# Supplementary material for: The genome of an apodid holothuroid (Chiridota heheva) provides insights into its adaptation to a deep-sea reducing environment
Source: Commun Biol. 2022 Mar 10;5:224. doi: 10.1038/s42003-022-03176-4 (PMC8913654; doi:10.1038/s42003-022-03176-4)
Supplement: Supplementary file 3 — Description of Additional Supplementary Files [file 42003_2022_3176_MOESM3_ESM.pdf]

Description of Additional Supplementary Files

File name: Supplementary Data 1
Description: Gene families that are expanded in C. heheva compared to other echinoderms.
